# Loss of 5-Hydroxymethylcytosine Is an Independent Unfavorable Prognostic Factor for Esophageal Squamous Cell Carcinoma

**DOI:** 10.1371/journal.pone.0153100

**Published:** 2016-04-06

**Authors:** Xuejiao Shi, Yue Yu, Mei Luo, Zhirong Zhang, Susheng Shi, Xiaoli Feng, Zhaoli Chen, Jie He

**Affiliations:** 1 Department of Thoracic Surgery, Cancer Hospital, Chinese Academy of Medical Sciences, Beijing, China; 2 Department of Pathology, Cancer Hospital, Chinese Academy of Medical Sciences, Beijing, China; Sapporo Medical University, JAPAN

## Abstract

Ten-eleven translocation (TET) enzymes catalyze the oxidation of 5-methylcytosine (5-mC) to 5-hydroxymethylcytosine (5-hmC), 5-formylcytosine and 5-carboxylcytosine, which result in genomic DNA demethylation. It was reported that 5-hmC levels were decreased in a variety of cancers and could be regarded as an epigenetic hallmark of cancer. In the present study, 5-hmC levels were detected by immunohistochemistry (IHC) in 173 esophageal squamous cell carcinoma (ESCC) tissues and 91 corresponding adjacent non-tumor tissues; DNA dot blot assays were used to detect the 5-hmC level in another 50 pairs of ESCC tissues and adjacent non-tumor tissues. In addition, the mRNA level of *TET1*, *TET2* and *TET3* in these 50 pairs of ESCC tissues was detected by real-time PCR. The IHC and DNA dot blot results showed that 5-hmC levels were significantly lower in ESCC tissues compared with corresponding adjacent non-tumor tissues (P = 0.029). *TET2* and *TET3* expression was also significantly decreased in tumor tissues compared with paired non-tumor tissues (*TET2*, P < 0.0001; *TET3*, P = 0.009), and the decrease in 5-hmC was significantly associated with the downregulation of *TET2* expression (r = 0.405, P = 0.004). Moreover, the loss of 5-hmC in ESCC tissues was significantly associated with poor overall survival among patients with ESCC (P = 0.043); multivariate Cox regression analysis showed that the loss of 5-hmC in ESCC tissues was an independent unfavorable prognostic indicator for patients with ESCC (HR = 1.569, P = 0.029). In conclusion, 5-hmC levels were decreased in ESCC tissues, and the loss of 5-hmC in tumor tissues was an independent unfavorable prognostic factor for patients with ESCC.

## Introduction

DNA cytosine methylation is one of the most important epigenetic modifications and is involved in various biological processes, such as genomic imprinting, X chromosome inactivation, and gene expression regulation. In the mammalian genome, almost all DNA methylation occurs at the C-5 atom of cytosine in CpG dinucleotides. 5-Methylcytosine (5-mC) is initially generated by the DNA methyltransferases Dnmt3a and Dnmt3b and is maintained by Dnmt1 during DNA replication [1]. Tahiliani et al. were the first to report that ten-eleven translocation (TET) enzymes, a family of Fe(2+)- and 2-oxoglutarate-dependent dioxygenases, catalyze the oxidation of 5-mC into 5-hydroxymethylcytosine (5-hmC), 5-formylcytosine (5-fC), and 5-carboxylcytosine (5-caC) and play a key role in DNA demethylation [2]. There are three *TET* genes in mammalian cells, *TET1*, *TET2* and *TET3*, and these genes are responsible for the tissue-dependent conversion of 5-mC to 5-hmC [3]. Several hematologic malignancies result from mutations in *TET2* [4,5], and a reduced level of 5-hmC has frequently been reported in association with *TET* mutations in myelodysplasia and leukemia [6,7]; furthermore, mutations in isocitrate dehydrogenase 1 and 2 (*IDH1/2*) lead to the production of 2-hydroxyglutarate (2-HG), which inhibits the activity of TET proteins [8,9].

In addition to hematologic malignancies, the dysregulation of TET2 or 5-hmC has been implicated in the carcinogenesis of several solid tumors. Lian et al. found that loss of 5-hmC is an epigenetic hallmark of melanoma with diagnostic and prognostic value; patients with lower 5-hmC levels had worse outcomes. Moreover, overexpression of active TET2 in human melanoma cells suppressed tumor growth in NOD/SCID xenograft mice and improved tumor-free survival, which suggested that the decrease in TET2 could be one of the mechanisms leading to the loss of 5-hmC in melanoma [10]. Furthermore, Kudo et al. reported decreased 5-hmC in 72.7% of colorectal tumor tissues and 75% of gastric tumor tissues [11]. Haffner et al. found that 5-hmC levels were lower in breast, prostatic and colorectal cancer compared with corresponding non-tumor tissues [12]. Other studies have obtained similar results in hepatic, lung and pancreatic cancer [13].

In the present study, we detected 5-hmC levels by IHC and DNA dot blot assays and *TET1/2/3* expression by real-time PCR in ESCC tissues. The clinical value of altered 5-hmC levels and the relationship between 5-hmC levels and *TET* expression were investigated.

## Materials and Methods

### Patients and tissue samples

Written informed consents were signed by all the patients enrolled in this study. This study was approved by the Institutional Review Board of the Cancer Hospital of the Chinese Academy of Medical Sciences and was conducted according to the guidelines approved by the ethics committee. We retrospectively enrolled 173 patients who underwent curative resection for ESCC at the Cancer Hospital of the Chinese Academy of Medical Sciences between December 2005 and December 2007. The formalin-fixed, paraffin-embedded specimens from these patients, including 173 tumor tissues and 91 adjacent non-tumor tissues, were obtained for immunohistochemical detection. Another 50 patients with ESCC who underwent curative resection at our hospital between April 2008 and June 2009 were enrolled in this study for the validation test. The frozen stored specimens corresponding to 50 pairs of tumor tissues and adjacent non-tumor tissues were obtained for DNA dot blot and real-time polymerase chain reaction (RT-PCR) assays. The demographic and clinicopathological information, including age, gender, tobacco use, alcohol use, differentiation grade, tumor location, T stage, lymph node metastasis, and pTNM stage, were reviewed for these 223 patients. The pTNM stage of ESCC was reclassified according to the seventh edition of the American Joint Committee on Cancer staging system. Follow-up information for 173 ESCC patients was obtained for the survival analysis.

### Immunohistochemistry

Briefly, tissue sections were deparaffinized, rehydrated, treated with 2N HCl for 15 min, and treated with 100 mM Tris-HCl, pH 8.5, for 10 min. Subsequently, the sections were dipped in a 3% hydrogen peroxide solution for 30 min and then incubated with goat serum at room temperature for 30 min. Then, the sections were incubated overnight with an anti-5-hmC antibody (1:5000 dilution; Active Motif, Cat # 39769, Carlsbad, CA, USA) at 4°C. After the sections were washed with PBS, they were incubated with polyclonal peroxidase-conjugated anti-rabbit IgG (Zhongshanjinqiao, Beijing, China) at room temperature for 20 min. Subsequently, DAB and hematoxylin were used to stain and counterstain the sections, respectively. Two pathologists independently calculated the IHC staining score by combining the percentage of positive cells and the staining intensity according to the following criteria: –, no cells stained; +, 1–40% of cells stained; ++; 40–70% of cells stained; and +++, 70–100% of cells stained. For the subsequent analysis,—was classified as negative 5-hmC expression, and + to ++ was classified as positive 5-hmC expression; no tissues were scored as +++.

### Dot blot assay

The dot blot assay procedure was modified from that described by Ito [14]. Briefly, genomic DNA was extracted from ESCC tissues or cultured cells using a DNeasy Blood and Tissue Kit (Qiagen, GmbH, Hilden, Germany). DNA was denatured by boiling at 100°C for 10 min, snap-freezing on ice, and incubation at -20°C for 20 min. Denatured genomic DNA (200 ng) was spotted onto a nitrocellulose filter membrane (Whan GmbH, Maidstone, Kent, UK), dried at room temperature, and then baked at 80°C for 30 min. The membrane was subsequently blocked in 5% non-fat milk for 2 h and incubated overnight with a primary antibody against 5-hmC (1:5000 dilution; Active Motif, Cat # 39769, Carlsbad, CA, USA) at 4°C. After three washes with TBST, the blots were incubated with polyclonal peroxidase-conjugated anti-rabbit IgG (Zhongshan jinqiao, Beijing, China) at room temperature for 1 h and subsequently developed using enhanced chemiluminescence (Millipore, Billerica, MA, USA) according to the manufacturer’s protocol. The signals were quantified using ImageJ software. In addition, another membrane was stained with 0.02% methylene blue in 0.3 M sodium acetate (pH 5.2) to visualize the DNA as a total genomic DNA loading control. The experiments were repeated for twice.

### Real-time polymerase chain reaction (RT-PCR)

Total RNA isolated from ESCC tissues or cultured cells using TRIzol reagent (Invitrogen, Carlsbad, CA, USA) was used to synthesize complementary DNA (cDNA) with a First Strand cDNA Synthesis Kit (Thermo Fisher Scientific, Rockford, IL, USA). Using SYBR Green PCR Master Mix (Invitrogen, Carlsbad, CA, USA), quantitative RT-PCR (qPCR) was conducted with an Applied Biosystems 7900 RT-PCR system (Applied Biosystems, Foster City, CA, USA) according to the manufacturer’s instructions. The 2^−ΔΔCt^ method was used to quantify expression levels, and *GAPDH* was used as an internal control. Three independent experiments were performed, and each sample was tested in triplicate. The primers used in the present study are shown in S1 Table.

### Statistical analysis

Statistical analyses were performed using SPSS 19.0 software. The χ^2^ test or paired t test was used to analyze differences in 5-hmC expression. The χ^2^ test or Fisher's exact test was used to analyze associations between 5-hmC expression and clinicopathological parameters. Pearson correlation analysis was used to evaluate the association between 5-hmC and *TET* mRNA expression. Kaplan-Meier curves and the log-rank test were used for the survival analysis, and univariate and multivariate Cox proportional hazards regression analyses were used to evaluate the risk factors for poor survival among patients with ESCC. Differences were considered significant at P < 0.05.

## Results

### Expression of 5-hmC was decreased in ESCC tissues and cell lines

**Fig 1:**
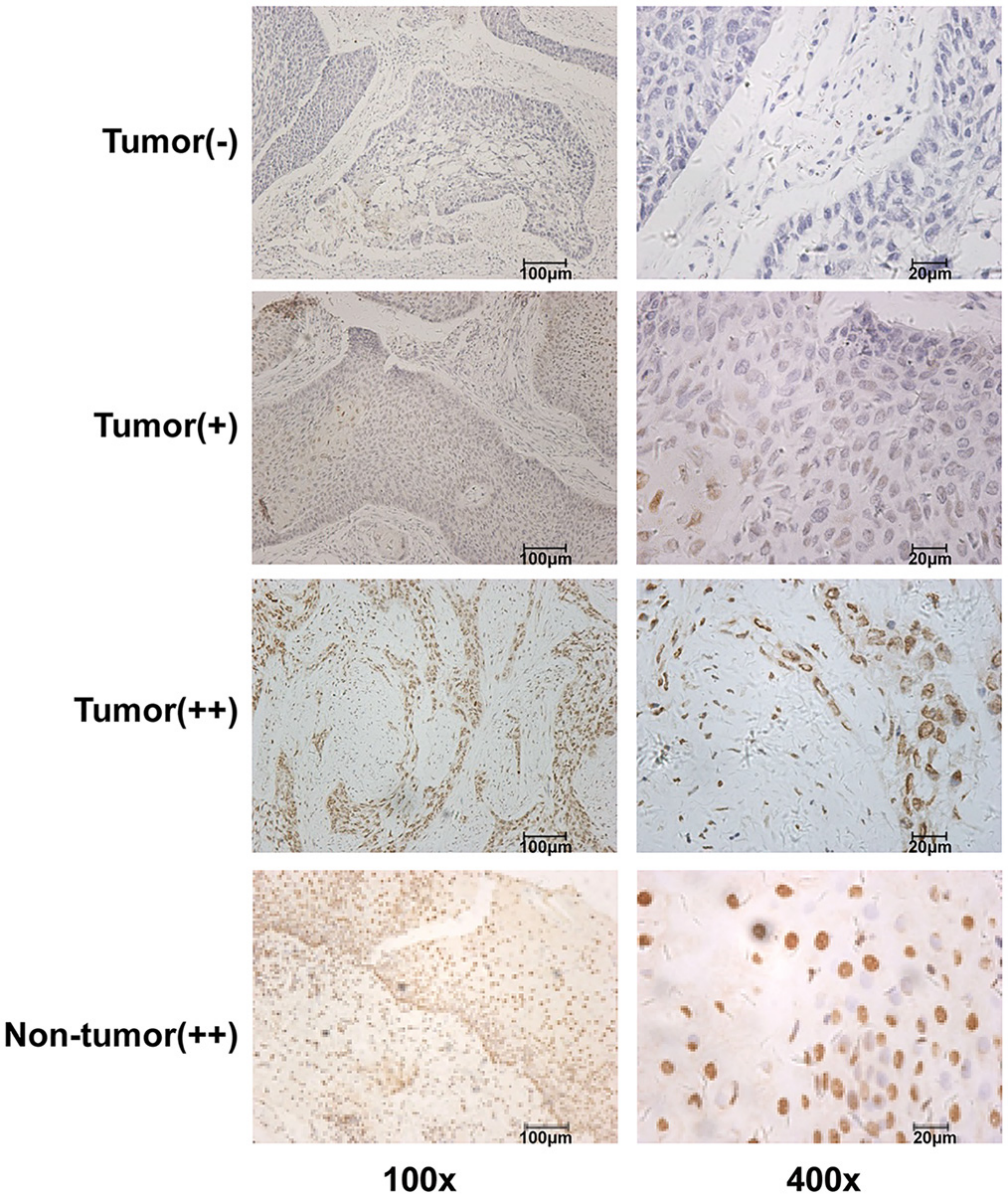
Expression of 5-hmC was decreased in ESCC tissues. Detection of 5-hmC expression in 173 ESCC tissues and 91 corresponding non-tumor tissues using IHC. Representative images are shown (left panel, 100×; right panel, 400×).

**Fig 2:**
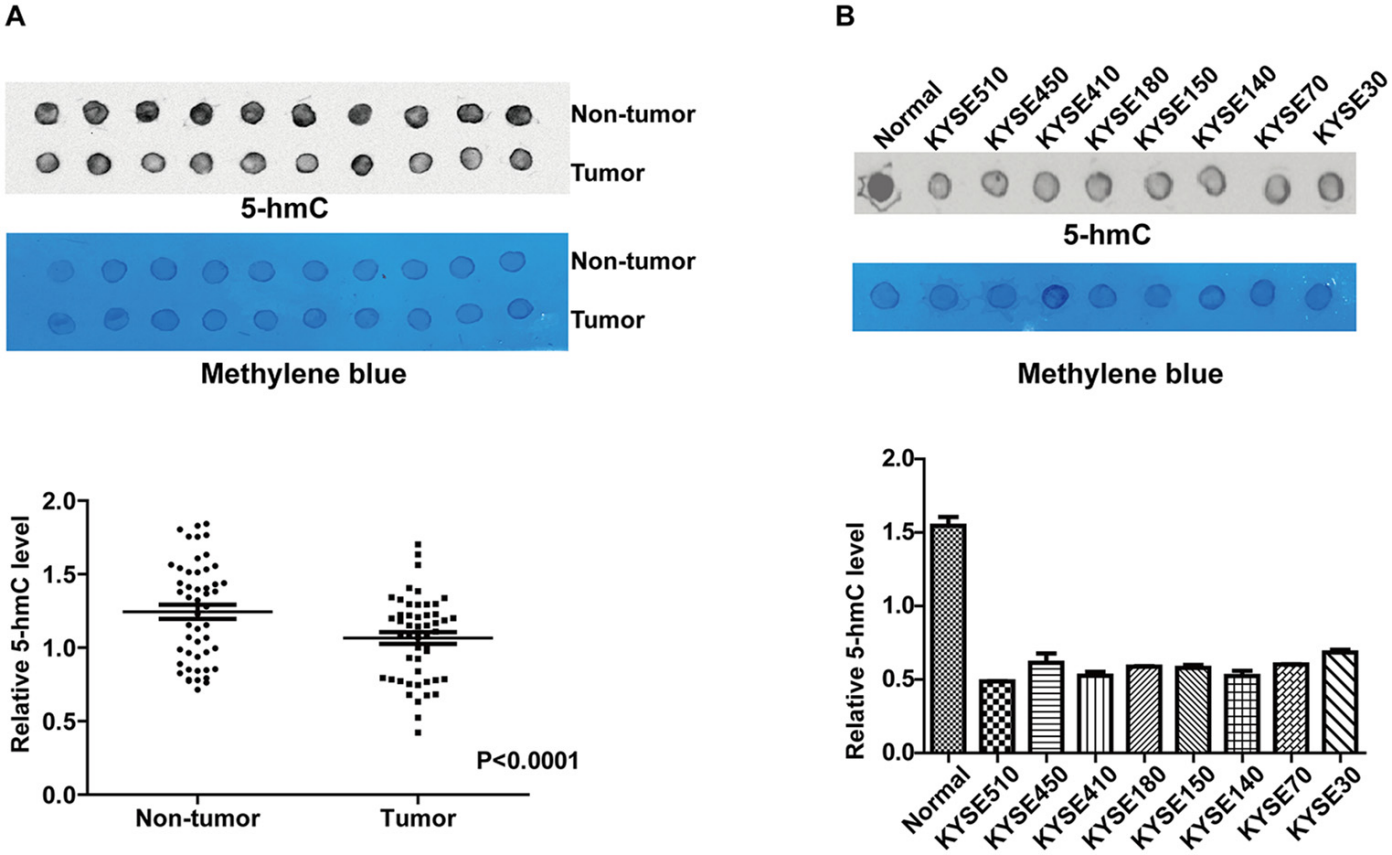
The expression of 5-hmC was decreased in ESCC tissues and cell lines. (A) Detection of 5-hmC expression in 50 matched ESCC tissues and corresponding non-tumor tissues using DNA dot blot assay. (B) Detection of 5-hmC level in 8 ESCC cell lines using DNA dot blot assay, the DNA isolated from 10 normal esophageal tissues were random selected and pooled as normal control. The methylene blue staining of 200 ng total genomic DNA was used as loading control. The intensity of the dot signal was quantified using ImageJ software, the relative 5-hmC level was calculated by dividing the value of 5-hmC by methylene blue, paired *t* test was used.

There were no differences in the clinicopathological characteristics between the 173- and 50-person cohorts of patients with ESCC in this study (Table 1). The immunohistochemistry results showed that positive 5-hmC staining was primarily located in the nucleus of tumor cells and esophageal epithelial cells (Fig 1), and that 5-hmC expression was significantly downregulated in ESCC tissues compared with adjacent non-tumor tissues. Overall, 62% (56/91) of non-tumor tissues showed positive 5-hmC expression, whereas 47% (82/173) of tumor tissues were positive for 5-hmC expression; this difference was statistically significant (P = 0.029; Table 2). Moreover, 5-hmC levels were detected by DNA dot blot assay in another 50 pairs of ESCC and adjacent non-tumor tissues and 8 cell lines. The results showed that 5-hmC levels were significantly decreased in tumor tissues compared with paired normal tissues (P < 0.0001; Fig 2A), 8 ESCC cell lines also showed markedly decreased 5-hmC level compared with normal tissue control (Fig 2B).

**Table 1:** Clinicopathological characteristics of patients with ESCC.

Characteristics	Cases for IHC (n = 173)		Cases for dot blot (n = 50)		*P*
	No.	%	No.	%	
**Age**					0.588
≥60	94	54.3	25	50	
<60	79	45.7	25	50	
**Gender**					0.864
Male	140	80.9	41	82.0	
Female	33	19.1	9	18.0	
**Tobacco use**					0.427
Yes	107	61.8	34	68.0	
No	66	38.2	16	32.0	
**Alcohol use**					0.204
Yes	104	60.1	35	70.0	
No	69	39.9	15	30.0	
**Tumor location**					0.212
Cervical/Upper	20	11.6	9	18.0	
Middle	87	50.3	28	56.0	
Lower	66	38.2	13	26.0	
**Histology grade**					0.327
G1	34	19.7	14	28.0	
G2	95	54.9	22	44.0	
G3-4	44	25.4	14	28.0	
**T stage**					0.200
T1	12	7.0	2	4.0	
T2	23	13.3	3	6.0	
T3	103	59.5	35	70.0	
T4	35	20.2	10	20.0	
**Lymph node metastasis**					0.317
N0	80	46.2	19	38.0	
N1	59	34.1	15	30.0	
N2	22	12.7	11	22.0	
N3	12	7.0	5	10.0	
**pTNM stage**					0.317
I	11	6.4	2	4	
II	72	41.6	16	32	
III	90	52.0	32	64	

χ^2^ test were used

**Table 2:** The IHC staining score in tumor and non-tumor tissues.

Scores	Tumor tissues (n = 173)		Non-tumor tissues (n = 91)		*P*
	No.	%	No.	%	
-	91	52.6	35	38.4	0.029
**+**	64	37.0	41	45.1	
**++**	18	10.4	15	16.5	

χ^2^ test were used

### Loss of 5-hmC was an independent prognostic factor of poor overall survival among patients with ESCC

**Fig 3:**
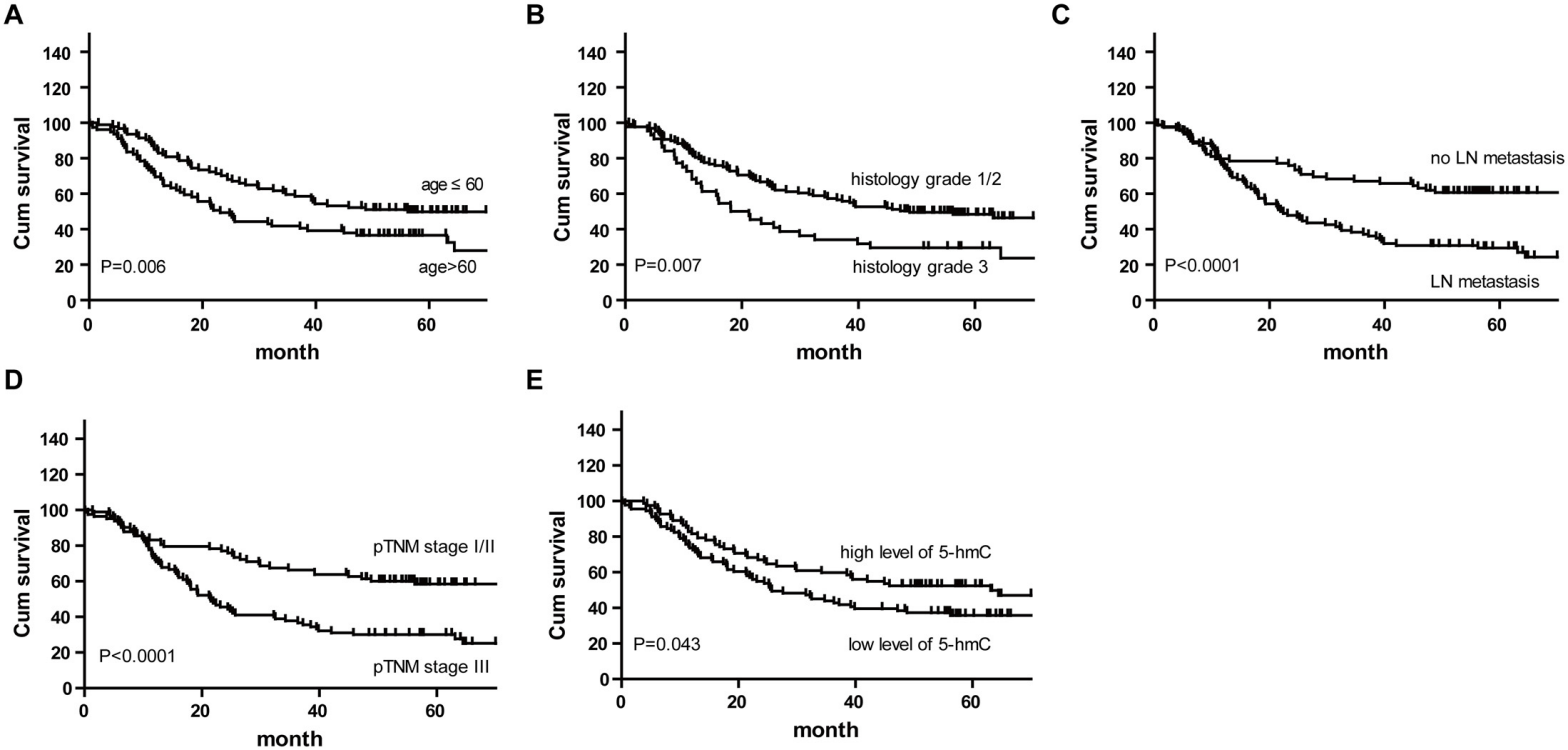
Associations between clinical parameters and overall survival. Kaplan-Meier analysis showed that older age (A), poor differentiation (B), lymph node metastasis (C), advanced pTNM stage (D) and negative 5-hmC expression (E) were significantly associated with poor overall survival among patients with ESCC.

Next, the relationships between 5-hmC expression in ESCC tissues and clinicopathological characteristics were analyzed in 173 patients. In this cohort, there were no significant associations between the loss of 5-hmC and clinicopathological characteristics such as age, gender, tobacco and alcohol use, tumor location, histology grade, lymph node metastasis and pTNM stage (S2 Table). Next, the survival of these 173 patients was analyzed, and the overall survival rate was determined to be 40.34%. The Kaplan-Meier and log-rank test analysis found that older age (P = 0.006), poor differentiation (P = 0.007), lymph node metastasis (P < 0.0001), advanced pTNM stage (P < 0.0001) and loss of 5-hmC (P = 0.043) were significantly associated with poor overall survival among patients with ESCC (Fig 3). Furthermore, the multivariate Cox proportional hazards regression analysis identified older age (HR = 1.650, P = 0.013), poor differentiation (HR = 1.718, P = 0.014), lymph node metastasis (HR = 2.150, P < 0.001) and loss of 5-hmC (HR = 1.569, P = 0.029) as independent prognostic predictors for patients with ESCC (Table 3).

**Table 3:** Univariate and multivariate analyses of overall survival among patients with ESCC.

	Univariate analysis		Multivariate analysis	
	HR (95% CI)	*P*	HR (95% CI)	*P*
**Age**				
(≥60 *vs* <60)	1.734 (1.161–2.589)	**0.007**	1.650 (1.110–2.451)	**0.013**
**Gender**				
(Male *vs* Female)	0.739 (0.459–1.188)	0.211		
**Tobacco use**				
(Yes *vs* No)	0.705 (0.474–1.049)	0.085		
**Alcohol use**				
(Yes *vs* No)	0.880 (0.591–1.310)	0.529		
**Histology grade**				
(G3 *vs* G1/2)	1.781 (1.167–2.714)	**0.007**	1.718 (1.116–2.644)	**0.014**
**T stage**				
(3/4 *vs* 1/2)	1.097 (0.665–1.810)	0.717		
**Lymph node metastasis**				
(Yes *vs* No)	2.325 (1.522–3.553)	**<0.001**	2.150 (1.402–3.297)	**<0.001**
**pTNM stage**				
(III *vs* I/II)	2.284 (1.509–3.454)	**<0.001**		
**5-hmC**				
(low *vs* high)	1.505 (1.010–2.244)	**0.045**	1.569 (1.048–2.351)	**0.029**

### Decreased 5-hmC expression was associated with *TET2* downregulation and gene mutation

**Fig 4:**
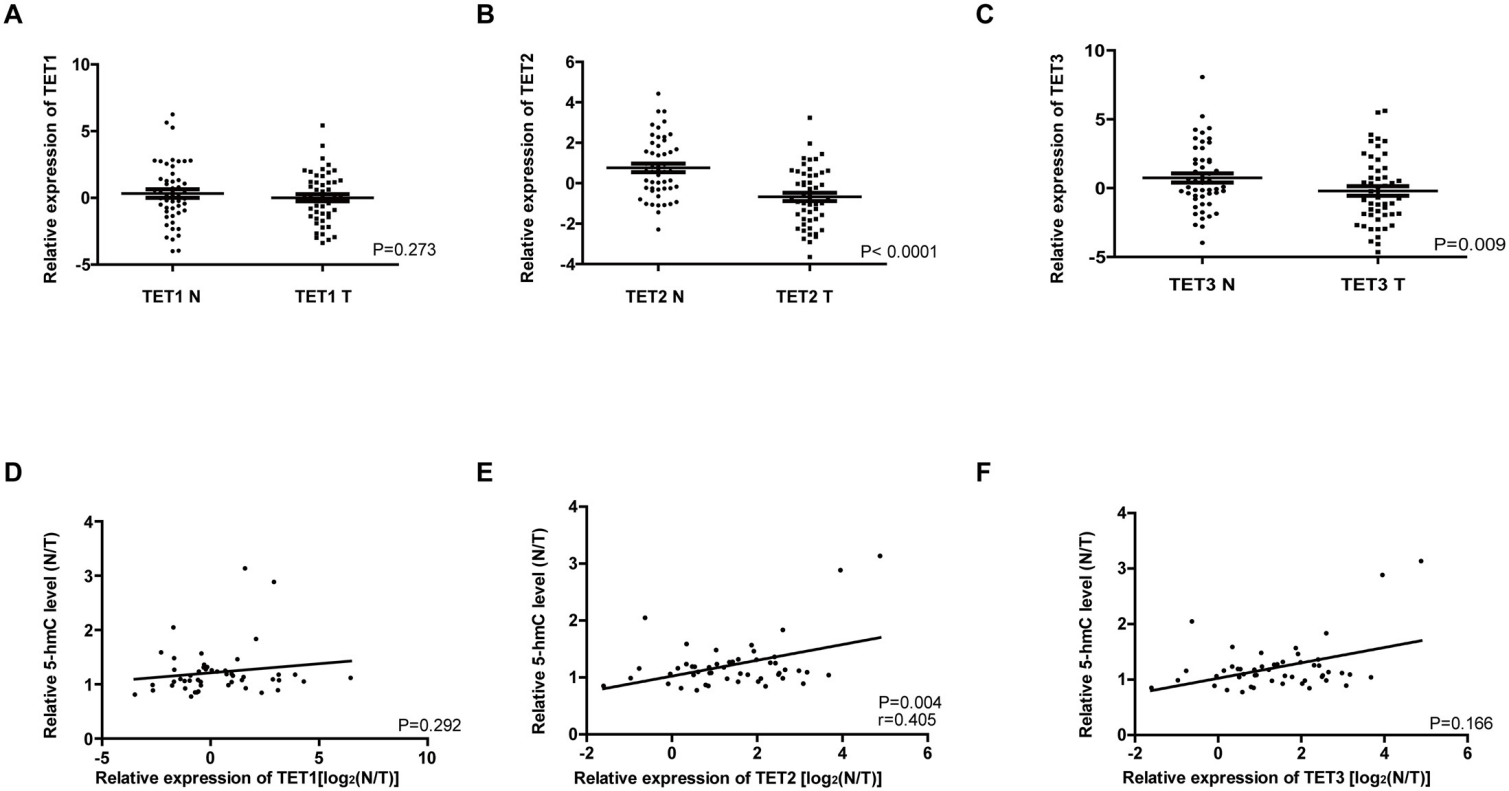
Decreased 5-hmC expression was associated with *TET2* downregulation. (A, B and C) *TET1/2/3* mRNA levels in 50 matched ESCC tissues and non-tumor tissues were compared. 2^−ΔΔCt^ method was used to calculate the relative expression of *TET*, and *GAPDH* was used as an internal control, the sample with the median value of *TET1/2/3* among 100 samples were used for normalization. The data were presented on a logarithmic scale. (D, E and F) Associations between *TET* level and 5-hmC levels in ESCC were analyzed. The relative 5-hmC level were calculated by N/T. The relative *TET* expression were calculated by 2^−ΔΔCt (N-T)^ and converted to a logarithmic scale on X axis.

TET catalyzes the oxidation of 5-mC into 5-hmC, and downregulation of TET, especially TET2, have been reported in human cancers [13,15]. Therefore, we measured the mRNA levels of three *TET* genes in the 50 pairs of ESCC tissues and adjacent non-tumor tissues. Compared with non-tumor tissues, *TET2* and *TET3* were significantly downregulated in ESCC tissues (P < 0.01) (Fig 4B and 4C), whereas *TET*1 expression showed no significant decrease in ESCC tissues (Fig 4A). In addition, *TET2* expression in tumor tissues was significantly associated with lymph node metastasis among patients with ESCC (P = 0.040; Table 4). Then, the relationship between *TET* downregulation and decreased 5-hmC levels was analyzed in these 50 patients with ESCC. The results showed that *TET2* downregulation was significantly associated with decreased 5-hmC levels in tumor tissues compared with non-tumor tissues (r = 0.405, P = 0.004; Fig 4E), however, altered expression of *TET1* or *TET3* was not associated with decreased 5-hmC levels (Fig 4D and 4F).

**Table 4:** Correlations between *TET2* levels in ESCC tissues and clinicopathological characteristics of patients with ESCC.

Characteristic	*TET2* expression		*P*
	Low	High	
**Age**			0.046
<60	17	8	
≥60	9	16	
**Gender**			0.281
Male	23	18	
Female	3	6	
**Tobacco use**			0.547
No	7	9	
Yes	19	15	
**Alcohol use**			0.902
No	8	7	
Yes	18	17	
**Location**			0.131
Cervical/Upper	2	7	
Middle	17	11	
Lower	7	6	
**Histology grade**			0.352
G1/G2	17	19	
G3	9	5	
**T stage**			0.661
T1/T2	2	3	
T3/T4	24	21	
**Lymph node metastasis**			**0.040**
No	6	13	
Yes	20	11	
**pTNM stage**			0.077
I/II	6	12	
III	20	12	

χ^2^ test were used

In addition to the downregulated expression of *TET* genes, it was reported that decreased 5-hmC expression in malignant tumors could be caused by mutations in the *TET*, *IDH1* or *IDH2* genes [5,8]. Whole exome sequencing data for 105 of the 173 cases with IHC staining in the present study were obtained from our previous study [16], and 12 of the 105 cases had mutations in the *TET1/2/3* or *IDH1/2* genes (Table 5). We analyzed potential correlations between *TET* or *IDH1* gene mutations and 5-hmC levels in these 105 cases. The result showed lower 5-hmC expression in patients with *TET* or *IDH1* mutations than in those with wild type versions of these genes, although the P value was not significant (P = 0.051; Table 6).

**Table 5:** The mutation of *TET* and *IDH1*gene in 105 ESCC tissues.

Case No.	Gene Symbol	Nucleotide (genomic, hg18)	Amino Acid (protein)	Mutation Type	Consequence	5-hmC level
422	IDH1	chr2_208812895–208812895_C_T	310G>R	Substitution	Missense	-
398	TET1	chr10_70120752–70120752_G_		Deletion	Frameshift	-
418	TET1	chr10_70074830–70074830_G_A	780E>K	Substitution	Missense	-
419	TET1	chr10_70121038–70121040_GAA_	1958E>	Deletion	In-frame deletion	-
410	TET1	chr10_70120599–70120599_A_G		Substitution	Silent	-
388	TET2	chr4_106376473–106376473_A_G	642Q>R	Substitution	Missense	-
411	TET2	chr4_106378020–106378020_G_T	1158D>Y	Substitution	Missense	+
442	TET2	chr4_106416109–106416109__GACC		Insertion	Frameshift	-
464	TET2	chr4_106383462–106383462_A_T	1175I>F	Substitution	Missense	-
464	TET2	chr4_106383463–106383463_T_A	1175I>N	Substitution	Missense	-
467	TET2	chr4_106376689–106376689_C_A	714S>X	Substitution	Nonsense	-
462	TET3	chr2_74181272–74181272_T_C		Substitution	Silent	-
409	TET3	chr2_74182677–74182677_G_T	1617A>S	Substitution	Missense	++

**Table 6:** The correlation between *TET* or *IDH1* gene mutations and 5-hmC expression.

Gene mutation	5-hmC expression		*P*
	Negative	Positive	
Yes	10	2	0.051
No	50	43	

## Discussion

DNA methylation is one of the most well-studied epigenetic modifications, and it has been demonstrated to play an important role in carcinogenesis [17]. In particular, accumulated methylation of CpG islands has been associated with alterations in chromatin organization, leading to changes in locus-specific transcriptional activity [18]. 5-mC is associated with gene repression and is regarded as a stable, highly heritable marker [19]. Recently, 5-hmC was identified as a novel epigenetic modification that was converted from 5-mC through the actions of TET family members [2]. The generation of 5-hmC is known to modulate 5-mC-dependent gene regulation, and 5-hmC is expressed in a tissue-dependent manner [3]. Previous studies have indicated that 5-hmC expression is decreased in multiple types of human cancers [13,15,12,10,20], and Murata et al. reported reduced 5-hmC and TET2 expression in esophageal cancer [21]. Consistent with these findings, the data obtained in the present study showed that the 5-hmC level was significantly reduced in ESCC tissues compared with non-tumor tissues using both IHC and DNA dot blot assays. Furthermore, we detected 5-hmC expression in 8 ESCC cell lines using DNA dot blot assays; 5-hmC was reduced in all these ESCC cell lines. These data indicated that the loss of 5-hmC expression is an epigenetic hallmark of ESCC. Although the hydrogen peroxide blocking might introduce a risk of artificial higher 5hmC level, as well as the quality of archival tissues could lead to reduced 5hmC level. All these factors created the systematic bias without affecting the result and conclusion of our study. Recent studies have demonstrated a correlation between the loss of 5-hmC expression and the outcome of patients with melanoma [10]. The data obtained in our study also suggested that the loss of 5-hmC was an independent unfavorable prognostic factor for patients with ESCC.

It was reported that reduced 5-hmC expression could result from decreased levels of TET1/2/3 [13]. In the present study, we measured *TET1/2/3* mRNA expression levels in 50 matched ESCC samples, compared with the corresponding non-tumor tissues, *TET2* and *TET3* gene expression was significantly reduced in tumor tissues, but downregulation of only *TET2* was significant associated with decreased 5-hmC, suggesting that decreased 5-hmC expression primarily reflects decreased *TET2* expression in ESCC. In our study, we have only found the significant correlation between the down-regulation of *TET2* and decreased 5-hmC when compared tumor tissues with normal tissues. The significant correlation of the 5-hmC level and TET2 expression in tumor tissues were not observed, we consider the possible reasons are the relative small samples and semiquantitative detection of 5-hmC. Unfortunately, we lack enough specimens for IHC staining in these 50 pairs of tissues. So, the correlation of the 5-hmC level and *TET2* expression in ESCC tumor tissues need to be further studied in a larger samples or using other method to detect the 5-hmC level.

In addition to the downregulated expression of *TET* genes, it was reported that decreased 5-hmC expression in malignant tumors could be caused by mutations in the *TET*, *IDH1* or *IDH2* genes [5,8]. The majority of *TET* gene mutations occur in hematologic malignancies, whereas *IDH1/2* mutations are predominantly detected in tumors of the central nervous system [5,22–24]. Whole exome sequencing data for 105 of the 173 cases were used to analyzed potential correlations between *TET* or *IDH1* gene mutations and 5-hmC levels in our study, we found that the cases with *TET* or *IDH1* mutations had lower 5-hmC expression than cases with wild type *TET* or *IDH1* gene. These data are consistent with the results obtained in hematologic malignancies [7]. However, only 12 of the 105 cases had mutations in the *TET1/2/3* or *IDH1/2* genes in ESCC, the future studies need to be done to identify other gene mutations involved in 5-hmC downregulation in ESCC, and to investigate the influence of mutation on the gene function.

## Supporting Information

S1 Table The primers used in the study. (XLSX)

S2 Table The relationships between 5-hmC level in tumor tissues and clinicopathological characteristics in 173 ESCC patients. (DOCX)
