# Supplementary material for: Loss of 5-Hydroxymethylcytosine Is an Independent Unfavorable Prognostic Factor for Esophageal Squamous Cell Carcinoma
Source: PLoS One. 2016 Apr 6;11(4):e0153100. doi: 10.1371/journal.pone.0153100 (PMC4822830; doi:10.1371/journal.pone.0153100)
Supplement: S2 Table — (DOCX) [file pone.0153100.s002.docx]

| **Characteristics** | **5-hmC positive (n=82)** | | **5-hmC negative (n=91)** | | ***P*** |
| --- | --- | --- | --- | --- | --- |
|  | **No.** | **%** | **No.** | **%** |  |
| **Age** |  |  |  |  | 0.659 |
| ≥60 | 46 | 56.1 | 48 | 52.7 |  |
| <60 | 36 | 43.9 | 43 | 47.3 |  |
| **Gender** |  |  |  |  | 0.890 |
| Male | 66 | 80.5 | 74 | 81.3 |  |
| Female | 16 | 19.5 | 17 | 18.7 |  |
| **Tobacco use** |  |  |  |  | 0.688 |
| Yes | 52 | 63.4 | 55 | 60.4 |  |
| No | 30 | 36.6 | 36 | 39.6 |  |
| **Alcohol use** |  |  |  |  | 0.475 |
| Yes | 47 | 57.3 | 57 | 62.6 |  |
| No | 35 | 42.7 | 34 | 37.4 |  |
| **Tumor location** |  |  |  |  | 0.282 |
| Cervical/Upper | 12 | 14.6 | 8 | 8.8 |  |
| Middle | 43 | 52.4 | 44 | 48.4 |  |
| Lower | 27 | 32.9 | 39 | 42.9 |  |
| **Histology grade** |  |  |  |  | 0.858 |
| G1 | 16 | 19.5 | 18 | 19.8 |  |
| G2/3/4 | 66 | 80.5 | 73 | 80.2 |  |
| **T stage** |  |  |  |  | 0.332 |
| T1 | 9 | 11.0 | 3 | 3.3 |  |
| T2 | 11 | 13.4 | 12 | 13.2 |  |
| T3 | 45 | 54.9 | 58 | 63.7 |  |
| T4 | 17 | 20.7 | 18 | 19.8 |  |
| **Lymph node metastasis** |  |  |  |  | 0.528 |
| N0 | 38 | 46.3 | 42 | 46.2 |  |
| N1 | 27 | 32.9 | 32 | 35.2 |  |
| N2 | 9 | 11.0 | 13 | 14.3 |  |
| N3 | 8 | 9.8 | 4 | 4.4 |  |
| **pTNM stage** |  |  |  |  | 0.747 |
| I | 4 | 4.9 | 7 | 7.7 |  |
| II | 36 | 43.9 | 36 | 39.6 |  |
| III | 42 | 51.2 | 48 | 52.7 |  |

**The relationships between 5-hmC level in tumor tissues and clinicopathological**

**characteristics in 173 ESCC patients**
